# Appointment of Dr. William Pepper

**Published:** 1842-05-14

**Authors:** 


Dr. William Pepper was, on Monday the 9th of May, elected one of the Physicians to the Pennsylvania Hospital, to fill the vacancy caused by the resignation of Dr. B. H. Coates. This is an excellent appointment, and gives general satisfaction.

The physicians to the hospital now are, Drs. Wood, Stewardson, and Pepper; the surgeons, Drs. Randolph, (still absent in Europe,) Norris and Edward Peace.

